# Fluctuations in serum lipid levels during neoadjuvant treatment as novel predictive and prognostic biomarkers for locally advanced breast cancer: a retrospective analysis based on a prospective cohort

**DOI:** 10.1186/s12944-024-02140-x

**Published:** 2024-08-22

**Authors:** Xinru Chen, Yingying Zhao, Yaohui Wang, Yumei Ye, Shuguang Xu, Liheng Zhou, Yanping Lin, Jingsong Lu, Wenjin Yin

**Affiliations:** Department of Breast Surgery, Renji Hospital, School of Medicine, Shanghai Jiaotong University, No. 1630 Dongfang Road, Shanghai, 200127 China

**Keywords:** Breast cancer, Lipid metabolism, Low-density lipoprotein, Body mass index, Prognosis

## Abstract

**Background:**

With increasing attention given to host-specific lipid metabolism status, it is of urgent need to identify lipid metabolism indices with predictive or prognostic value in locally advanced breast cancer patients treated with neoadjuvant chemotherapy (NAC), and to evaluate the performance improvement by incorporating them into the existing Neo-Bioscore staging system.

**Methods:**

Patients from a prospectively maintained database of locally advanced breast cancer patients who received radical surgery after NAC between January 2014 to December 2020 were enrolled in this study. The enrolled patients were randomly divided into a training set and a test set at a ratio of 6:4. The random forest algorithm was applied to rank the importance of prognostic factors, top-ranked lipid metabolism indices of which were then incorporated into Neo-Bioscore to construct an updated prognostic model. The performances of these two models were compared in both training set and test set from multiple perspectives. Study outcomes included disease-free survival (DFS), relapse-free survival (RFS), distance-recurrence-free survival (DRFS), locoregional-recurrence-free survival (LRFS) and overall survival (OS).

**Results:**

A total of 200 eligible patients were included in this study. After a median follow-up of 4.73 years, it was demonstrated that the relative increase in total cholesterol (TC; DFS: HR = 4.782, 95%CI 1.410 ~ 16.217, *P* = 0.012) and low-density lipoprotein (LDL; DFS: HR = 4.622, 95%CI 1.517 ~ 14.088, *P* = 0.007) during NAC led to poorer survival outcomes. Patients with either a higher body mass index (BMI) or elevated LDL during NAC had a worse prognosis (DFS: HR = 6.351, 95%CI 1.938 ~ 20.809, *P* = 0.002; OS, HR = 6.919, 95%CI 1.296 ~ 36.932, *P* = 0.024). Incorporating BMI and LDL fluctuations during NAC into Neo-Bioscore improved the prognostic stratification, especially in terms of LRFS (*P* = 0.046 vs. *P* = 0.65) and OS (*P* = 0.013 vs. *P* = 0.61). Multidimensional evaluation confirmed the improvement in model fit and clinical use for the updated model in both training set and test set.

**Conclusions:**

This is the first study to illustrate the relative elevation of LDL and TC levels during NAC as independent prognosticators for locally advanced breast cancer. This is also the first attempt to incorporate lipid metabolism indices into the original Neo-Bioscore staging system, which further improves the prognostic stratification of patients receiving NAC.

**Supplementary Information:**

The online version contains supplementary material available at 10.1186/s12944-024-02140-x.

## Background

Neoadjuvant chemotherapy (NAC) occupies an increasingly vital position in the whole-course management of breast cancer, which is due to its advantages in shrinking tumors, making down-staging possible, and eliminating micro-metastatic foci prior to surgery. NAC is capable of rendering inoperable patients amenable to curative surgery, thus increasing the likelihood of breast conserving surgery, and even improving the survival outcomes of patients [1, 2]. In addition, NAC provides a platform for the early evaluation of tumor sensitivity to certain cytotoxic agents in vivo, which offers considerable guidance for tailoring subsequent strategies. It is well known that achieving pathological complete response (pCR) signifies an improved long-term survival benefit [3]. To more precisely stratify the prognosis of breast cancer patients who underwent NAC, specific prognostic scoring systems have been established. The Neo-Bioscore staging system, which evolved from the CPS + EG staging system, encompasses pretreatment clinical stage, posttreatment pathological stage, and tumor biological markers (estrogen receptor [ER], human epidermal growth factor receptor 2 [HER2], and grade), and has been recognized as being an excellent algorithm for assessing the prognosis of patients treated with NAC [4]. However, Neo-Bioscore merely includes the intrinsic characteristics of the tumor, whereas the patient's own metabolic factors have gradually become valuable. Accordingly, it is worthwhile to investigate whether Neo-Bioscore can be further optimized by incorporating the host's characteristics of a specific internal microenvironment.

Previous studies have demonstrated that in certain types of cancers, a high-fat diet, obesity and hyperlipidemia are not only closely related to the tumor initiation and growth [5–7], but are also strongly associated with increased metastatic capacity [8–10]. These findings indicate that lipid metabolism plays a preponderant role in the occurrence and development of multiple cancers.

The process by which tumor cells constantly adjust metabolic patterns to facilitate reproduction and adapt to the tumor microenvironment (TME) is generally referred to as "metabolic reprogramming", which is a novel hallmark of cancer [11, 12]. Lipids, as one of the major sources of energy, indispensable components of biofilms, and signaling molecules required for intra- and extracellular transmission, are key ingredients in metabolic rewiring [13–16]. Studies have shown that "lipid metabolism reprogramming" has a bearing on proliferation, invasion, energy generation, plasma membrane remodeling, oncogenic signal propagation, and chemotherapy resistance [17]. In an effort to meet the demand of excessive lipids, tumor cells regulate specific enzymes to boost the endogenous synthesis of lipids while also taking up exogenous lipids from the TME and peripheral circulating blood [18]. Although "lipid metabolism reprogramming" in breast cancer has been intensively studied in terms of both endogenous synthesis and the TME, few reports have clarified the relationship between serum lipids and patient prognosis in breast cancer patients receiving NAC [19, 20].

On these premises, this study was performed to explore the correlation of serum lipid levels at baseline (pre-NAC) or before surgery (post-NAC) and their fluctuations during NAC with the efficacy and prognosis for breast cancer patients who received NAC. Moreover, this study aimed to identify lipid metabolism indices with predictive/prognostic value, and incorporated them into the Neo-Bioscore staging system to evaluate the reliability and feasibility of the updated model.

## Methods

### Patients

**Fig. 1:**
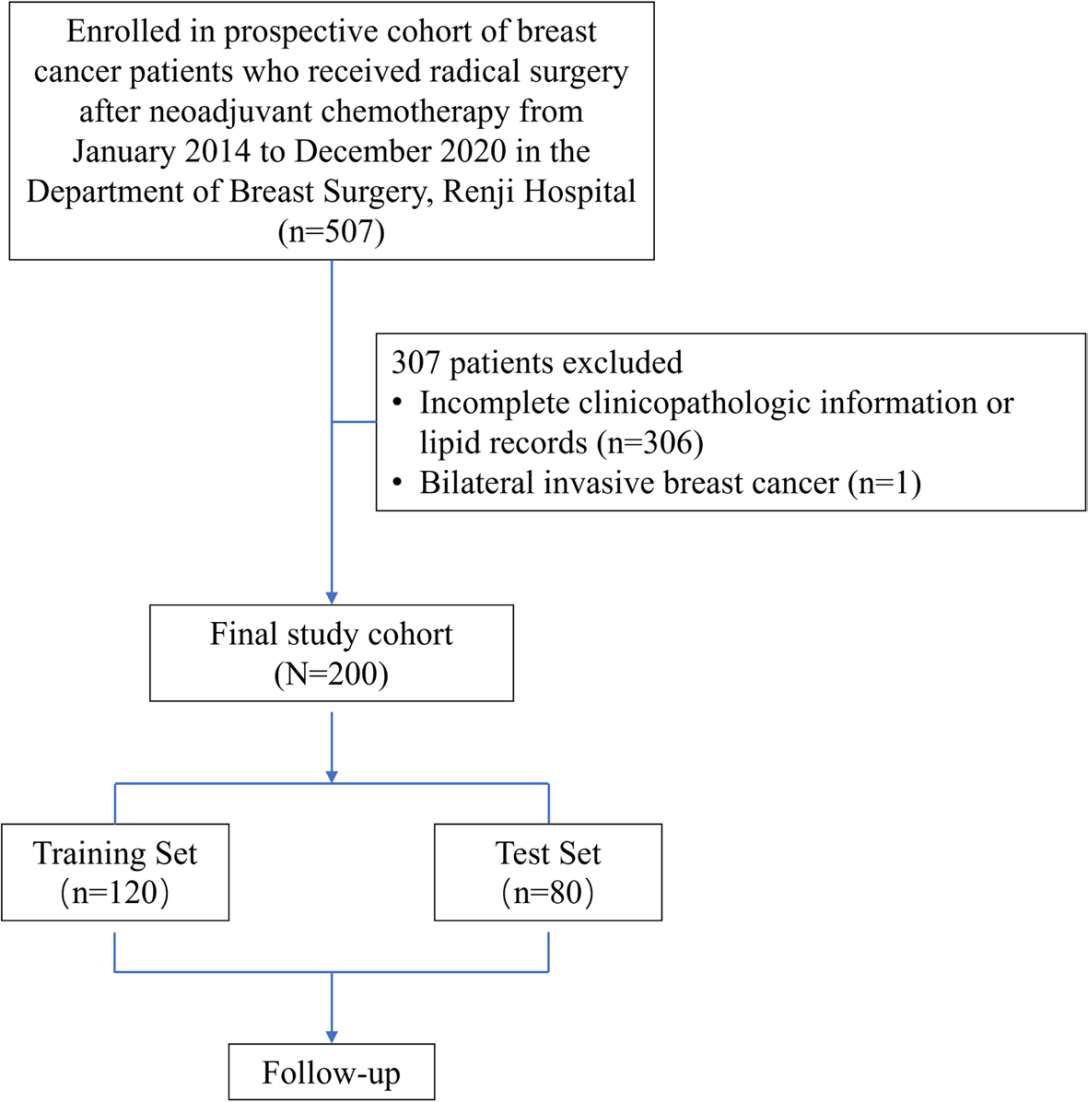
Data collection Abbreviations: NAC, neoadjuvant chemotherapy

Patients were retrospectively recruited from a prospectively maintained database (NCT 05621564) who underwent radical surgery after NAC between January 2014 and December 2020 at the Department of Breast Surgery, Renji Hospital, School of Medicine, Shanghai Jiaotong University (Fig. 1). Patients were eligible if they were females aged 18 years or older with pathologically confirmed invasive breast cancer (T1 N1-3 or T2-4 N0-3, M0), available clinicopathological information and serum lipid level (at least pre-NAC and post-NAC) records. The key exclusion criteria included the administration of only endocrine therapy in the neoadjuvant setting, metastatic breast cancer and bilateral invasive breast cancer. This study was conducted in accordance with the Reporting Recommendations for Tumor Marker Prognostic Studies (REMARK) statement [21] and the protocol was approved by the Independent Ethics Committee of Renji Hospital with approval number of LY2022-028-B.

### Collection and processing of clinicopathological information

The clinicopathological information (including age, height, weight, menopausal status, pathological information, and clinical stage, among other information) of eligible breast cancer patients was prospectively collected at baseline (pre-NAC). Body mass index (BMI), which is one of the key indicators reflecting systemic lipid metabolism, was calculated as weight (kg) divided by the square of height (m). Hormone receptor status was considered to be positive if there were at least 1% positive tumor nuclei in the immunohistochemical (IHC) staining of either the ER or progesterone receptor (PR), and ER positivity was defined as at least 1% positive tumor nuclei in the IHC staining of the ER. In accordance with the 2018 recommendations of the American Society of Clinical Oncology/College of American Pathologists [22], HER2 was determined as positive for tumors of IHC 3 + or IHC 2 + with amplification by fluorescence in situ hybridization (FISH). Patients were followed up through outpatient visits or telephone interviews every 3 months in the first 2 years, every 6 months until the fifth year and then annually until death or any relapse.

### Serum lipid levels

The serum lipid biomarker data, including triglyceride (TG), total cholesterol (TC), high-density lipoprotein (HDL), low-density lipoprotein (LDL) and non-high-density lipoprotein (NHDL) levels, were prospectively measured at baseline (pre-NAC) and before surgery (post-NAC). The fluctuation of serum lipids was obtained by subtracting the baseline serum lipid level (pre-NAC) from the preoperative serum lipid level (post-NAC).

### Data separation and cross-validation

The enrolled patients were randomly divided into a training set and a test set at a ratio of 6:4. The test set was isolated for the final test for the prognostic models. For the training set, serum lipids with predictive and prognostic value were identified and K-fold cross validation was utilized based on Cox regression, wherein the training group (two-thirds of the training set) and validation group (one-third of the training set) were randomly generated, and the process was repeated 5 times to ensure the fitting degree of the original Neo-Bioscore model and the updated model.

### Identification and incorporation of lipid metabolism indices

Random forest algorithm (ggRandomForests R package v2.2.1) was applied to rank the importance of prognostic factors in the Neo-Bioscore staging system (clinical stage, pathological stage, ER status, HER2 status and histological grade) as well as lipid metabolism indices (BMI, TG, TC, HDL, LDL, and NHDL) through the variable importance (VIMP) method and minimum depth method in the training set for estimating disease-free survival (DFS) and relapse-free survival (RFS). The top-ranked markers were then merged into the Neo-Bioscore staging system to construct an updated prognostic model (Neo-Bioscore + Lipid Metabolism).

### Performance evaluation of prognostic models

The performances of the original model (Neo-Bioscore) and the updated model (Neo-Bioscore + Lipid Metabolism) were compared with those of multidimensional methods, including time-dependent receiver operating characteristic (ROC) curves and their area under the curve (AUC), decision curve analysis (DCA), the Akaike information criterion (AIC), the C-index, and Integrated Discrimination Improvement (IDI).

### Validation with an external database

A detailed gene set (HP_INCREASED_LDL_CHOLESTEROL_CONCENTRATION) related to increased serum lipid concentrations was identified via Gene Set Enrichment Analysis (GSEA) (http://www.gsea-msigdb.org/gsea/msigdb/index.jsp). Afterwards, Kaplan–Meier plotter [Breast Cancer] (http://kmplot.com/analysis/index.php) was applied to verify the relationship between the expression of these genes and the prognosis of breast cancer patients.

### Statistical analysis

The study outcomes were pCR, DFS, RFS, distance-recurrence-free survival (DRFS), locoregional-recurrence-free survival (LRFS) and overall survival (OS). Specifically, pCR was referred to as ypT0 ypN0; DFS was defined as the time from surgery to the first occurrence of local recurrence, regional recurrence, distant recurrence, second primary cancer, or death from any cause, and RFS was defined as the time from surgery to the first occurrence of local recurrence, regional recurrence, distant recurrence, or death from any cause. Similarly, the definition of DRFS is the period from surgery to the first occurrence of distant recurrence, and death from any cause, whereas the definition of LRFS is the period from surgery to the first occurrence of local recurrence, regional recurrence, and death from any cause. OS denoted the interval from surgery to death from any cause.

Continuous variables were compared by using *t* tests or Wilcoxon tests where appropriate. Categorical variables were compared by applying the chi-square or the Yates correction where appropriate. Correlations between variables are presented in the form of heatmaps. The best cutoffs of BMI and serum lipids were determined by using maximally selected rank statistics via the surv_cutpoint function in the survminer R package (v0.4.9). The associations between various factors and pCR were tested by using logistic regression. Kaplan–Meier analysis was performed to estimate survival outcomes, and survival differences between the groups were evaluated by using the log-rank test. Furthermore, a multivariate Cox proportional hazards regression model was used to estimate the hazard ratio (HR) and 95% confidence intervals (CIs). Adjustment factors for multivariate logistic and multivariate Cox regression analysis included ER status (positive vs. negative), HER2 status (positive vs. negative), Ki-67 level (> 30% vs. ≤ 30%) and clinical stage (II stage vs. III stage). Cox-based nomogram plots were used to show the contribution of each influencing factor in the model to survival outcomes. All of the statistical tests were two-sided, with *P* < 0.05 indicating statistical significance. The analyses were carried out in the R programming language (v4.2.2).

## Results

### Baseline clinicopathological characteristics

**Fig. 2:**
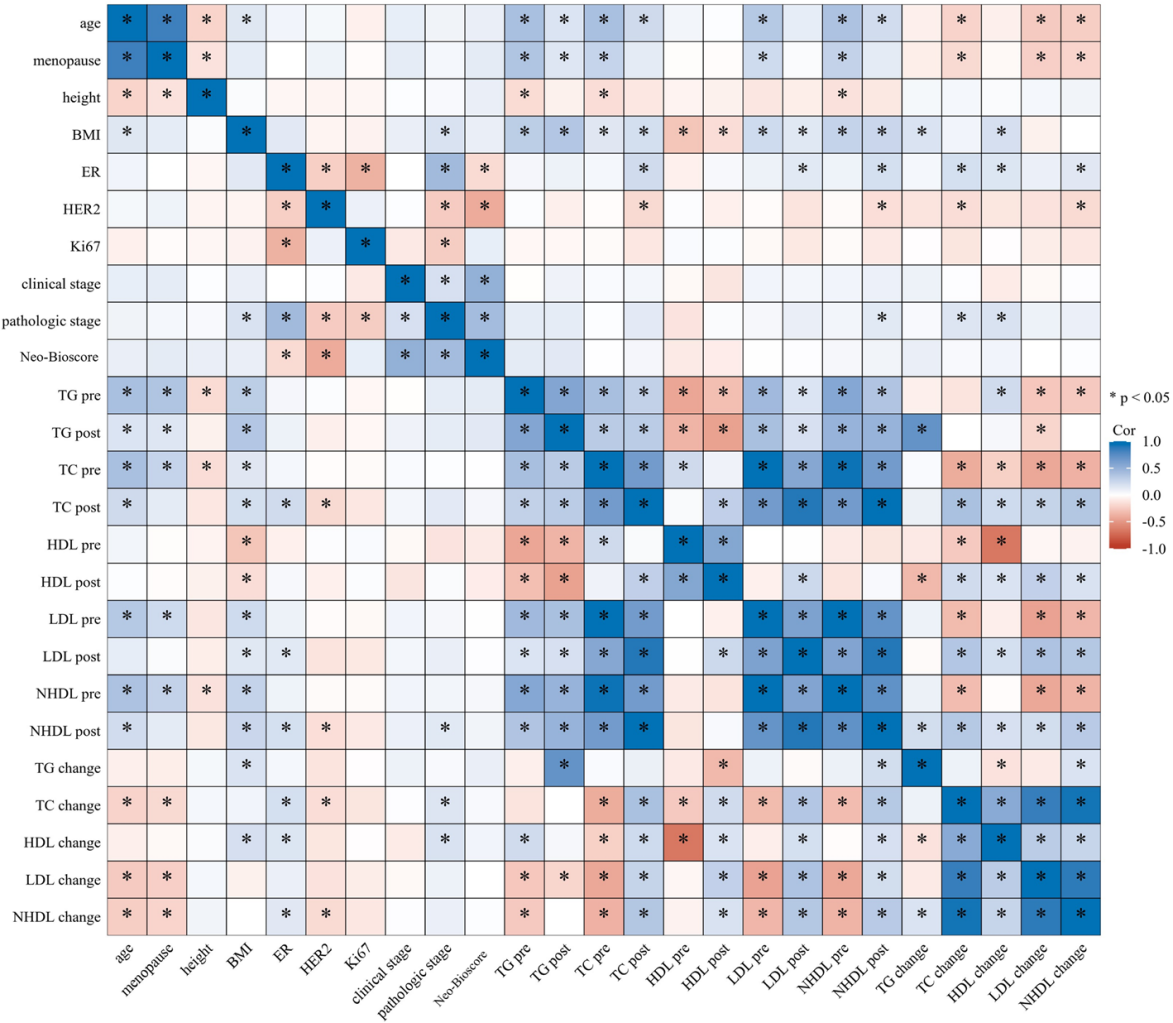
Correlation heatmap of clinicopathological parameters and serum lipid levels (pre-NAC, post-NAC and changes during NAC) Abbreviations: BMI, body mass index; ER, estrogen receptor; HER2, human epidermal growth factor receptor 2; TG, triglyceride; TC, total cholesterol; HDL, high-density lipoprotein; LDL, low-density lipoprotein; NHDL, non-high-density lipoprotein; pre, pre-neoadjuvant chemotherapy; post, post-neoadjuvant chemotherapy

A total of 200 eligible patients were included in this study. The median age was 52 years (range 25 ~ 71 years) and most of the tumors were luminal-like (n = 154, 77%). Patients with a high BMI (BMI > 25 kg/m^2^) accounted for 22.5% of the overall population. The vast majority of the patients had cT2-4 (*n* = 198, 99.0%) or cN1-3 (*n* = 168, 84.0%) tumors. The correlations between clinicopathological parameters and serum lipid levels were detailed in Fig. 2. The entire study population was randomly divided into a training set and a test set in a 6:4 ratio, which presented a well-balanced distribution of clinicopathological features in general (Table 1; Supplementary Table S1). As of March 31, 2023, 11 patients had died, 24 experienced DFS events and 20 experienced RFS events after a median follow-up of 4.73 years (range 1.68 ~ 7.06 years). The specific numbers of events for different outcomes in the training set and the test set were described in detail in Supplementary Table S2.

**Table 1:** Baseline clinicopathological characteristics

	**Overall (*N* = 200)**	**Training Set (*N* = 120)**	**Test Set (*N* = 80)**	***P* value**
Age (years), median (range)	52 (25–71)	52 (26–70)	52 (25–71)	0.57
Height (cm), median (range)	160 (148–176)	160 (148–170)	160 (150–176)	0.53
Weight (kg), median (range)	59 (37–87.5)	58.8 (37–82)	59 (45–87.5)	0.97
BMI (kg/m^2^), median (range)	23.21 (15.79–34.18)	23.23 (15.79–29.38)	23.07 (17.97–34.18)	0.89
Ki-67 level (%), median (range)	40 (2–90)	40 (3–90)	30 (2–80)	0.23
**Baseline serum lipid level (mmol/L), median (range)**				
TG	1.12 (0.14–4.88)	1.10 (0.14–4.88)	1.14 (0.37–3.37)	0.19
TC	4.61 (2.41–8.97)	4.58 (2.72–8.28)	4.64 (2.41–8.97)	0.75
HDL	1.32 (0.61–2.63)	1.38 (0.61–2.50)	1.26 (0.64–2.63)	0.04
LDL	2.73 (1.04–6.08)	2.69 (1.25–5.68)	2.77 (1.04–6.08)	0.82
NHDL	3.21 (1.47–7.82)	3.14 (1.67–6.81)	3.28 (1.47–7.82)	0.83
**Preoperative serum lipid level (mmol/L), median (range)**				
TG	1.56 (0.54–16.50)	1.48 (0.54–16.50)	1.77 (0.58–9.14)	0.01
TC	4.37 (2.07–8.07)	4.36 (2.07–8.07)	4.38 (2.12–7.71)	0.99
HDL	0.97 (0.36–1.69)	0.98 (0.49–1.67)	0.95 (0.36–1.69)	0.14
LDL	2.57 (0.49–5.00)	2.59 (1.11–5.00)	2.56 (0.49–4.35)	0.61
NHDL	3.42 (1.42–7.41)	3.38 (1.88–7.41)	3.43 (1.42–6.92)	0.80
**Menopausal status, N (%)**				
Premenopausal	97 (48.5)	59 (49.2)	38 (47.5)	0.82
Postmenopausal	103 (51.5)	61 (50.8)	42 (52.5)	
**Clinical T stage, N (%)**				
1	2 (1.0)	1 (0.8)	1 (1.3)	0.72
2	47 (23.5)	31 (25.8)	16 (20.0)	
3	82 (41.0)	46 (38.4)	36 (45.0)	
4	69 (34.5)	42 (35.0)	27 (33.7)	
**Clinical N stage, N (%)**				
0	32 (16.0)	19 (15.8)	13 (16.3)	0.84
1	121 (60.5)	73 (60.8)	48 (60.0)	
2	26 (13.0)	14 (11.7)	12 (15.0)	
3	21 (10.5)	14 (11.7)	7 (8.7)	
**Clinical stage, N (%)**				
II	52 (26.0)	32 (26.7)	20 (25.0)	0.79
III	148 (74.0)	88 (73.3)	60 (75.0)	
**pCR, N (%)**				
pCR	46 (23.0)	28 (23.3)	18 (22.5)	0.89
non-pCR	154 (77.0)	92 (76.7)	62 (77.5)	
**ER status, N (%)**				
Negative	65 (32.5)	46 (38.3)	25 (31.3)	0.31
Positive	135 (67.5)	74 (61.7)	55 (68.7)	
**HER2 status, N (%)**				
Negative	124 (62.0)	79 (65.8)	45 (56.3)	0.17
Positive	76 (38.0)	41 (34.2)	35 (43.7)	
**Subtype, N (%)**				
Hormone receptor-positive and HER2-negative	94 (47.0)	58 (48.3)	36 (45.0)	0.45
Hormone receptor-positive and HER2-positive	60 (30.0)	33 (27.5)	27 (33.7)	
Hormone receptor-negative and HER2-positive	16 (8.0)	8 (6.7)	8 (10.0)	
Triple negative	30 (15.0)	21 (17.5)	9 (11.3)	

*Abbreviations*: *BMI* Body mass index, *TG* Triglyceride, *TC* Total cholesterol, *HDL* High-density lipoprotein, *LDL* Low-density lipoprotein, *NHDL* Non-high-density lipoprotein, *T* Tumor, *N* Nodal, *pCR* Pathological complete response, *ER* Estrogen receptor, *HER2* Human epidermal growth factor receptor 2

### Correlation of serum lipid levels with pCR in the training set

With regard to baseline (pre-NAC) and preoperative (post-NAC) serum lipid levels, higher TC at baseline was found to be significantly correlated with a higher pCR rate in both univariate (OR = 2.879, 95%CI 1.073 ~ 7.720, *P* = 0.036) and multivariate analyses (OR = 3.033, 95%CI 1.031 ~ 8.923, *P* = 0.044; Supplementary Table S3). On the other hand, the relative increase in TC during NAC was associated with a lower rate of pCR (OR = 0.248, 95%CI 0.087 ~ 0.708, *P* = 0.009). Besides, the change in TC during NAC remained to be an independent predictor of pCR after multivariate adjustment (OR = 0.300, 95%CI 0.098 ~ 0.916, *P* = 0.035; Supplementary Table S4).

### Correlation of serum lipid levels with prognosis in the training set

**Fig. 3:**
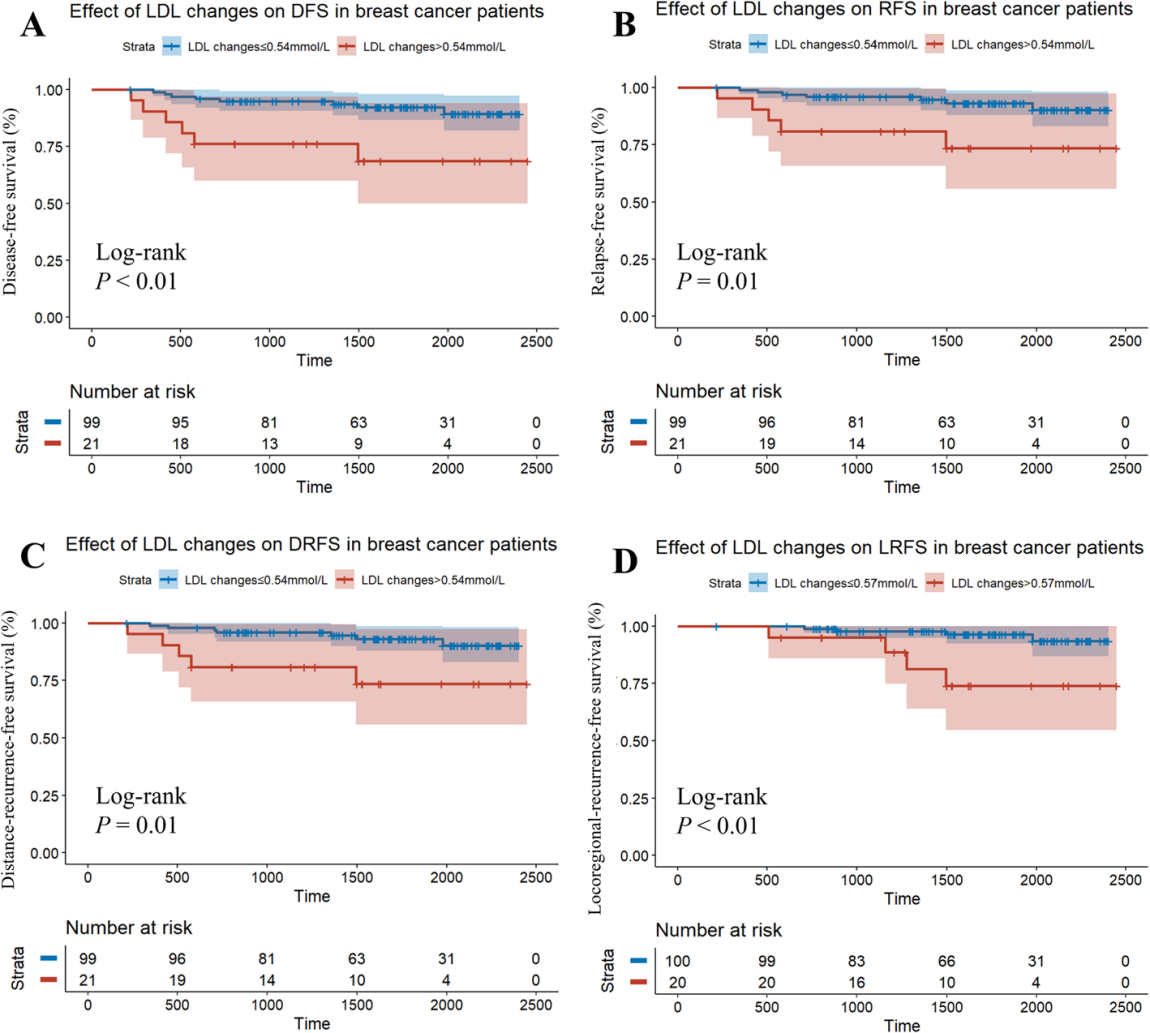
Kaplan–Meier curves for various survival outcomes by LDL changes during neoadjuvant chemotherapy in training set Kaplan–Meier estimates of LDL changes on (**A**) DFS, (**B**) RFS, (**C**) DRFS and (**D**) LRFS Abbreviations: DFS, disease-free survival; RFS, relapse-free survival; DRFS, distance-recurrence-free survival; LRFS, locoregional-recurrence-free survival; LDL, low-density lipoprotein

Although pre-NAC (Supplementary Figure S1) and post-NAC (Supplementary Figure S2) lipid levels were not significantly correlated with either DFS or RFS in the training set, a potential connection was discerned between the changes in lipid levels during NAC and various survival outcomes. Wherein, the Kaplan–Meier curves indicated that a relative increase in LDL led to significantly poorer DFS (*P* < 0.01; Fig. 3A), RFS (*P* = 0.01; Fig. 3B), DRFS (*P* = 0.01; Fig. 3C), and LRFS (*P* < 0.01; Fig. 3D). Additionally, the relative increase in TC (DFS: *P* < 0.01; Supplementary Fig. 3B) during the neoadjuvant period was significantly related to a worse prognosis.

Multivariate Cox regression analysis revealed the detrimental impact of relatively increasing TC (HR = 4.782, 95%CI 1.410 ~ 16.217, *P* = 0.012) and LDL (HR = 4.622, 95%CI 1.517 ~ 14.088, *P* = 0.007) on DFS (Table 2).

**Table 2:** Multivariate Cox proportional hazards model of lipid changes predicting DFS and RFS in training set

Lipid change	Survival outcome	Hazard ratio	95% confidence intervals	*P* value
**TG change**	DFS	0.906	0.117 ~ 7.012	0.925
	RFS	0.834	0.179 ~ 3.885	0.817
**TC change**	DFS	4.782	1.410 ~ 16.217	0.012*
	RFS	3.375	0.986 ~ 11.551	0.053
**HDL change**	DFS	1.718	0.479 ~ 6.168	0.407
	RFS	0.940	0.201 ~ 4.402	0.937
**LDL change**	DFS	4.622	1.517 ~ 14.088	0.007**
	RFS	4.832	1.401 ~ 16.664	0.013*

The adjustment factors included estrogen receptor status (positive vs. negative), HER2 status (positive vs. negative), Ki-67 level (> 30% vs. ≤ 30%) and clinical stage (II vs. III). *Abbreviations*: *DFS* Disease-free survival, *RFS* Relapse-free survival, *TG* Triglyceride, *TC* Total cholesterol, *HDL* High-density lipoprotein, *LDL* Low density lipoprotein

^*^P < 0.05, **P < 0.01

### Identification of lipid metabolism indices with prognostic value

**Fig. 4:**
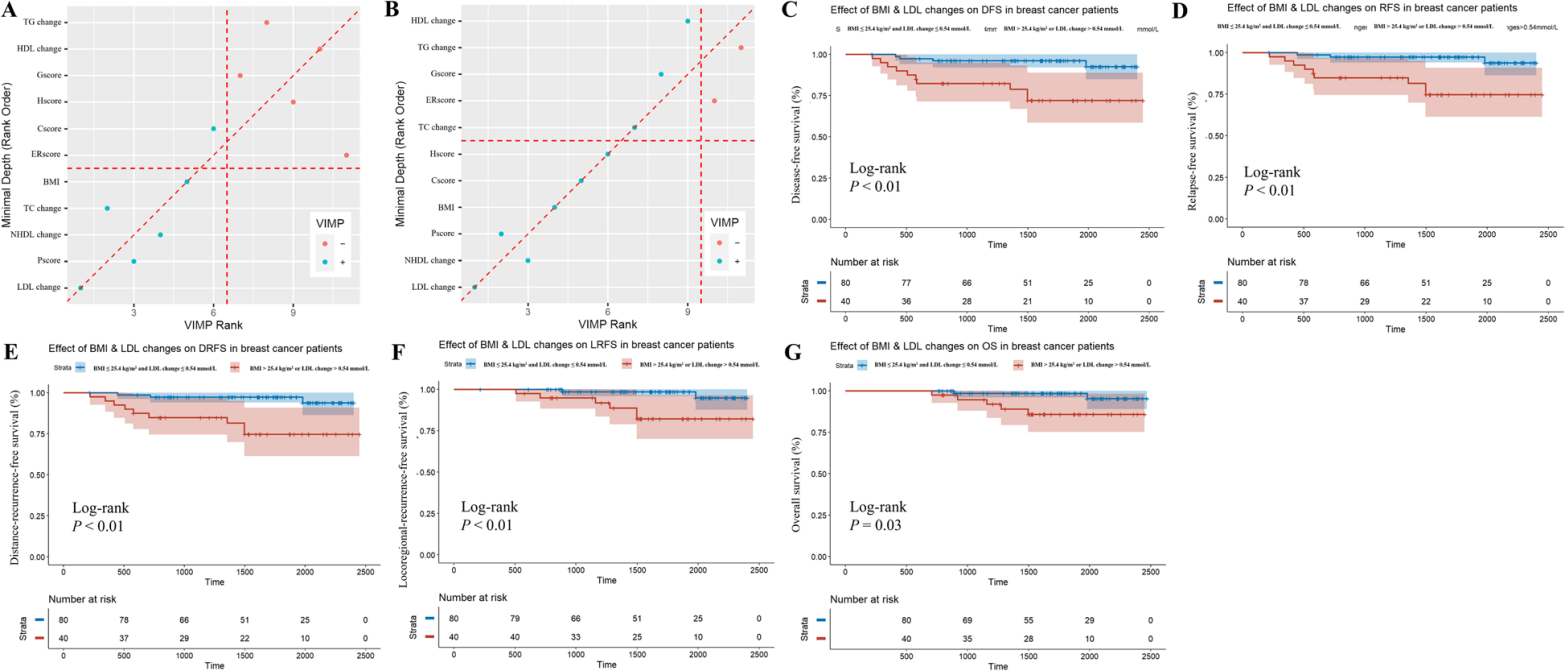
Identification of lipid metabolism indices with prognostic value in training set Random Forest applied to rank the importance of variables in accordance with efficiency in predicting (**A**) DFS and (**B**) RFS. Kaplan–Meier estimates of BMI and LDL changes on (**C**) DFS; (**D**) RFS; (**E**) DRFS; (**F**) LRFS; (**G**) OS Note: As for Fig. 4A & B, the horizontal axis is the importance ranking based on the VIMP method, with decreasing importance from left to right. VIMP < 0 (red points) indicates that the variable reduces the predictive accuracy, while VIMP > 0 (blue points) indicates that the variable improves the predictive accuracy. The vertical axis is used to sort the variable importance through minimal depth method, and the importance decreases from bottom to top. The closer it is to the bottom left corner, the higher ranking of the variable in both screening methods Abbreviations: DFS, disease-free survival; RFS, relapse-free survival; DRFS, distance-recurrence-free survival; LRFS, locoregional-recurrence-free survival; OS, overall survival; BMI, body mass index; Cscore, Clinical staging score in Neo-Bioscore; Pscore, Pathological staging score in Neo-Bioscore; ERscore, ER status score in Neo-Bioscore; Hscore, HER2 status score in Neo-Bioscore; Gscore, grade score in Neo-Bioscore; VIMP, variable importance

The importance of lipid metabolism indices was sorted by random survival forest in the training set, which indicated that BMI and LDL changes were invariable in the top rank in terms of predicting both DFS (Fig. 4A) and RFS (Fig. 4B). Kaplan–Meier curves further revealed that patients with either a high BMI or elevated LDL during NAC had inferior DFS (*P* = 0.0011; Fig. 4C), RFS (*P* = 0.0011; Fig. 4D), DRFS (*P* = 0.0011; Fig. 4E), LRFS (*P* = 0.0099; Fig. 4F) and OS (*P* = 0.026; Fig. 4G). After multivariate adjustment, the unfavorable effect of higher BMI or elevated LDL on prognosis remained significant (DFS: HR = 6.351, 95%CI 1.938 ~ 20.809, *P* = 0.002; RFS: HR = 7.743, 95%CI 2.038 ~ 29.421, *P* = 0.003; DRFS: HR = 7.730, 95%CI 2.034 ~ 29.370, *P* = 0.003; LRFS: HR = 9.965, 95%CI 1.909 ~ 52.015, *P* = 0.006; OS: HR = 6.919, 95%CI 1.296 ~ 36.932, *P* = 0.024).

### Optimization of prognostic model

**Fig. 5:**
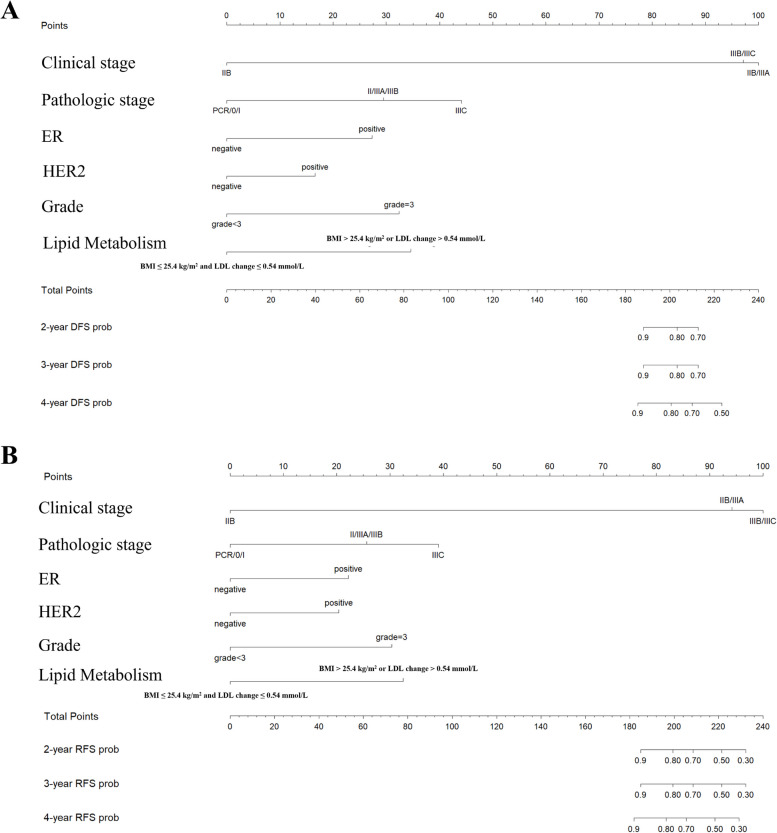
Nomograms of the updated model in predicting DFS (**A**) and RFS (**B**) in training set Abbreviations: DFS, disease-free survival; RFS, relapse-free survival; ER, estrogen receptor; HER2, human epidermal growth factor receptor 2

**Fig. 6:**
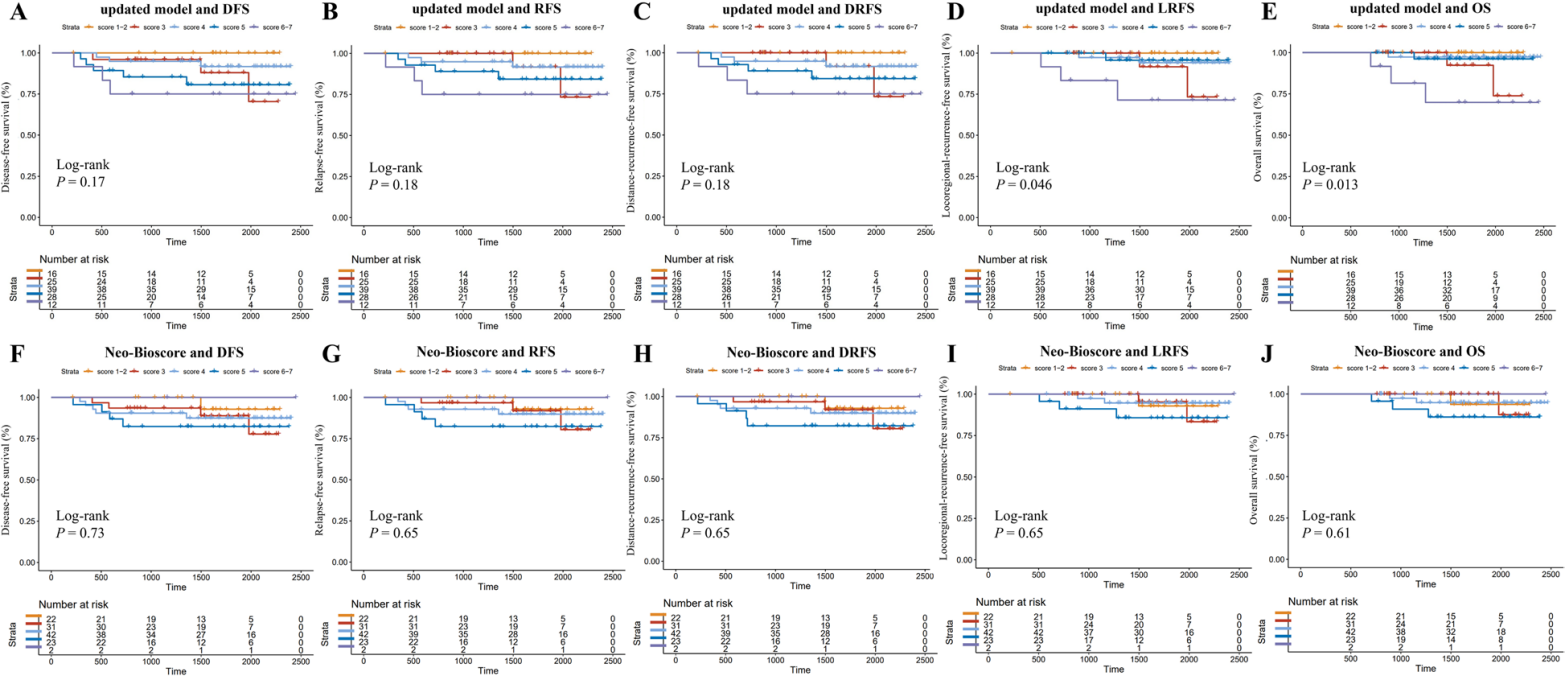
Prognostic stratification ability of the Neo-Bioscore and the updated model in training set Prognostic stratification of DFS (**A**, **F**), RFS (**B**, **G**), DRFS (**C**, **H**), LRFS (**D**, **I**), and OS (**E**, **J**) based on the updated model (**A** ~ **E**) and the Neo-Bioscore (**F** ~ **J**) in the training set. No patients scored 8 in updated model Abbreviations: DFS, disease-free survival; RFS, relapse-free survival; DRFS, distance-recurrence-free survival; LRFS, locoregional-recurrence-free survival; OS, overall survival

BMI and LDL fluctuations during NAC were incorporated into Neo-Bioscore as a representative indicators of lipid metabolism. For patients with a higher BMI or elevated LDL during NAC, an extra one point was added on the basis of the original Neo-Bioscore staging system (Table 3). The nomograms of the updated model (Neo-Bioscore + Lipid Metabolism) in the training set for DFS (Fig. 5A) and RFS (Fig. 5B) indicated that clinical stage, pathological stage and lipid metabolism index were the three most significant factors affecting the prognosis of patients receiving NAC. Besides, the updated model was more capable of stratifying patient prognosis than was Neo-Bioscore (Fig. 6), especially in terms of LRFS (*P* = 0.046 vs. *P* = 0.65, respectively; Fig. 6D, I) and OS (*P* = 0.013 vs. *P* = 0.61, respectively; Fig. 6E, J).

**Table 3:** Scoring rules for the Neo-Bioscore and the updated model

	**Neo-Bioscore**	**Neo-Bioscore + Lipid Metabolism**
**Clinical Stage**		
I	0	0
IIA	0	0
IIB	1	1
IIIA	1	1
IIIB	2	2
IIIC	2	2
**Pathological Stage**		
0	0	0
I	0	0
IIA	1	1
IIB	1	1
IIIA	1	1
IIIB	1	1
IIIC	2	2
**Tumor Markers**		
ER-negative	1	1
Grade 3	1	1
HER2-negative	1	1
**Lipid Metabolism**^a^		1

^a^The scoring principle for lipid metabolism was that one point was added when the patient reached one of two risk factors: BMI > 25.4 kg/m^2^ or LDL increased by more than 0.54 mmol/L during neoadjuvant chemotherapy

### Performance comparison between the Neo-Bioscore and the updated model in the training set and test set

**Fig. 7:**
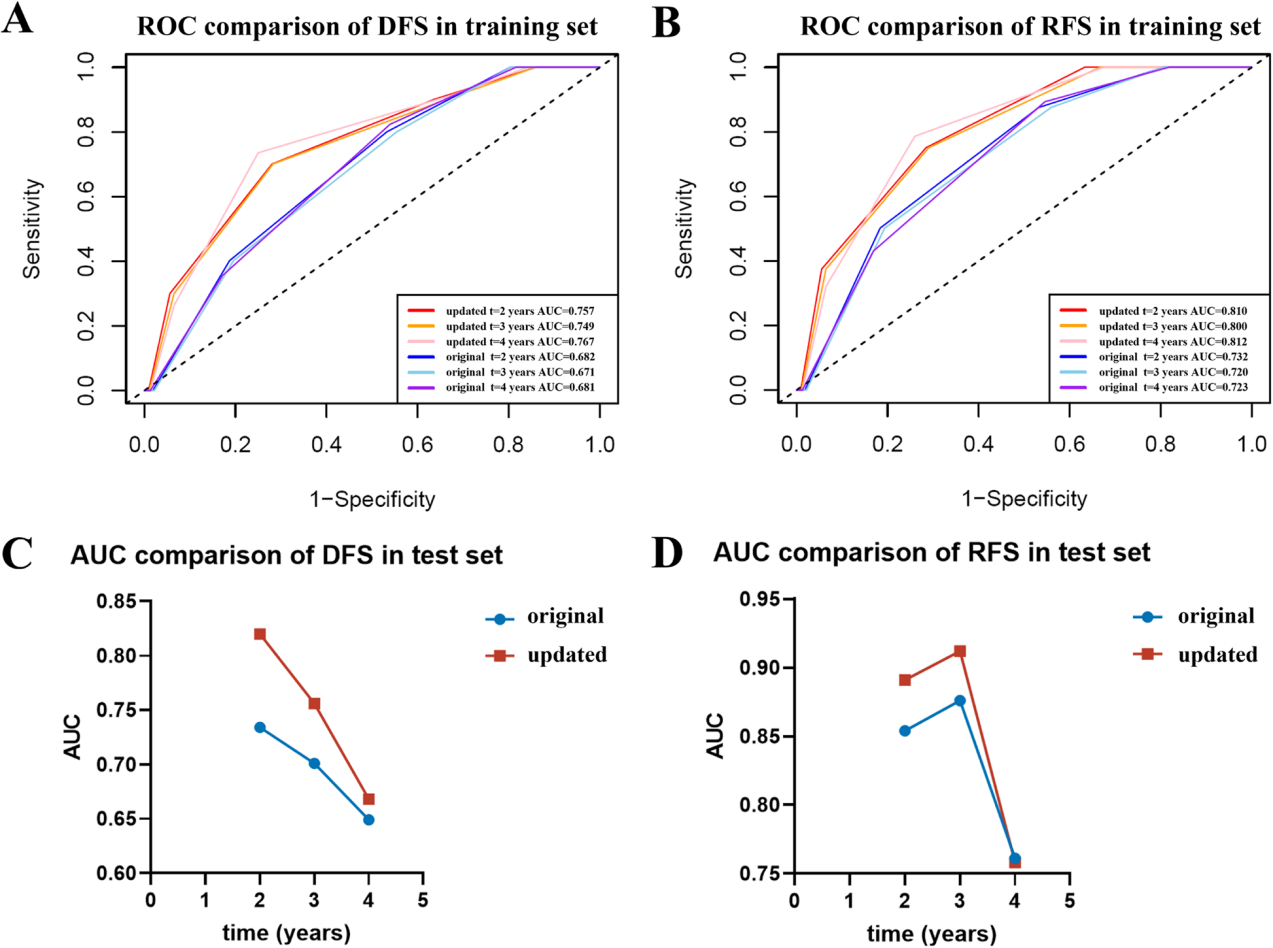
Time-dependent ROC curves and AUC in training set and test set Time-dependent ROC comparison between Neo-Bioscore (blue series) and updated model (red series) in the training set (**A**, **B**) and AUC comparison in the test set (**C**, **D**) in terms of DFS (**A**, **C**) and RFS (**B**, **D**) Abbreviations: ROC, receiver operating characteristic; AUC, area under curve; DFS, disease-free survival; RFS, relapse-free survival

**Fig. 8:**
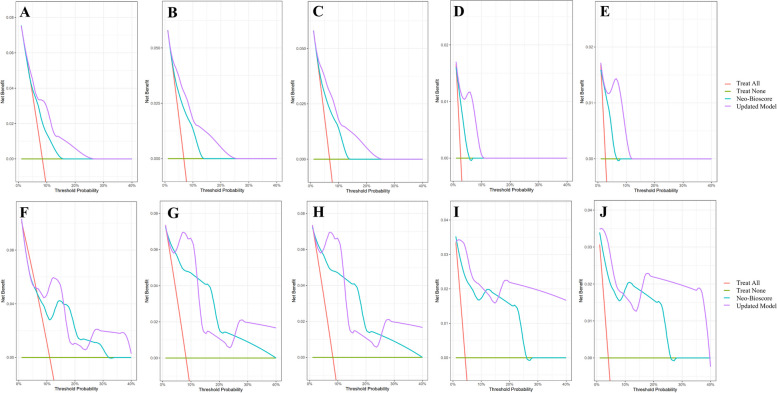
Decision Curve Analysis of Neo-Bioscore and the updated model in training set and test set Decision Curve Analysis of 3-year DFS (**A**, **F**), 3-year RFS (**B**, **G**), 3-year DRFS (**C**, **H**), 3-year LRFS (**D**, **I**) and 3-year OS (**E**, **J**) in the training set (**A** ~ **E**) and the test set (**F** ~ **J**) Abbreviations: DFS, disease-free survival; RFS, relapse-free survival; DRFS, distance-recurrence-free survival; LRFS, locoregional-recurrence-free survival; OS, overall survival

In the training set, the time-dependent ROC curves of the updated model (red series) were always greater than those of Neo-Bioscore (blue series). Regardless of the analysis time, the AUC of the updated model was greater than that of Neo-Bioscore in terms of both DFS (Fig. 7A) and RFS (Fig. 7B), thus indicating that the sensitivity and specificity of the updated model were superior to those of the original model. Furthermore, threefold 5-times cross validation was carried out in the training set (Table 4), which verified the superiority of the performance of the updated model to that of Neo-Bioscore. A similar conclusion was obtained in the test set (Fig. 7C, 7D). The DCA also displayed an improvement in the clinical net benefit of the updated model in both training set and test set (Fig. 8). After incorporating the lipid metabolism index into Neo-Bioscore, the AIC value in the training set decreased (DFS: 128.96 to 125.06; RFS: 109.94 to 105.79; Table 5), and the C-index significantly increased (DFS: 0.644 to 0.749, *P* = 0.001; RFS: 0.680 to 0.790, *P* = 0.003; Table 5). Moreover, the IDI significantly improved the predictive ability of the updated model. In addition, similar conclusions were drawn when the AIC, C-index and IDI of the two models were compared in the test set (Table 5).

**Table 4:** 3-fold 5-times cross validation of the performance between Neo-Bioscore and updated model in training set

	**AUC in the training set**		**AUC in threefold 5-times cross validation**	
	**Neo-Bioscore**	**Neo-Bioscore + Lipid Metabolism**	**Neo-Bioscore**	**Neo-Bioscore + Lipid Metabolism**
**3 years DFS**	0.671	0.749↑	0.651	0.758↑
**4 years DFS**	0.681	0.767↑	0.650	0.766↑
**3 years RFS**	0.720	0.800↑	0.718	0.832↑
**4 years RFS**	0.723	0.812↑	0.716	0.836↑

*Abbreviations*: *DFS* Disease-free survival, *RFS* Relapse-free survival, *AUC* Area under curve

**Table 5:** Akaike information criterion^a^, C-index^b^ and Integrated Discrimination Improvement^c^ comparison of Neo-Bioscore and the updated model

	**Training Set**			**Test Set**		
	**Neo-Bioscore**	**Neo-Bioscore + Lipid Metabolism**	***P* value**	**Neo-Bioscore**	**Neo-Bioscore + Lipid Metabolism**	***P* value**
**AIC DFS**	128.96	125.06↓	/	81.65	80.52↓	/
**AIC RFS**	109.94	105.79↓	/	59.72	59.19↓	/
**C-index DFS**	0.644	0.749↑	0.001	0.724	0.753↑	0.217
**C-index RFS**	0.680	0.790↑	0.003	0.879	0.864	0.779
**IDI 2-year DFS**	0.034 (0.002 ~ 0.120)		0.016	0.017 (-0.019 ~ 0.078)		0.363
**IDI 3-year DFS**	0.033 (0.002 ~ 0.118)		0.020	0.029 (-0.044 ~ 0.107)		0.419
**IDI 4-year DFS**	0.038 (0.003 ~ 0.132)		0.012	0.022 (-0.047 ~ 0.095)		0.527
**IDI 2-year RFS**	0.037 (0.002 ~ 0.133)		0.032	0.018 (-0.033 ~ 0.101)		0.563
**IDI 3-year RFS**	0.036 (0.002 ~ 0.132)		0.032	0.031 (-0.062 ~ 0.128)		0.527
**IDI 4-year RFS**	0.041 (0.003 ~ 0.139)		0.028	0.024 (-0.063 ~ 0.111)		0.647

*Abbreviations*: *DFS* Disease-free survival, *RFS* Relapse-free survival

^a^The Akaike information criterion (AIC) was utilized to compare the accuracy of the models. A more superior the model corresponded to a lower AIC value

^b^The C-index was used to assess the prediction accuracy of the model by evaluating the consistency between the predicted survival outcomes and the actual outcomes. The range of the C-index is [0.5–1]. If the prediction is completely inconsistent with the reality, then the C-index is 0.5. Otherwise, if the predicted outcomes are completely identical to reality, the C-index is equal to 1

^c^Integrated Discrimination Improvement (IDI) reflects the difference in the predicted probabilities between the updated and original models, which is calculated based on the prediction probability of each individual according to the established model. In general, a higher IDI corresponded to a better prediction efficiency of the new model. An IDI > 0 represents positive improvement. An IDI < 0 represents negative improvement. If IDI = 0, it is considered that there is no improvement

### Validation with an external database

**Fig. 9:**
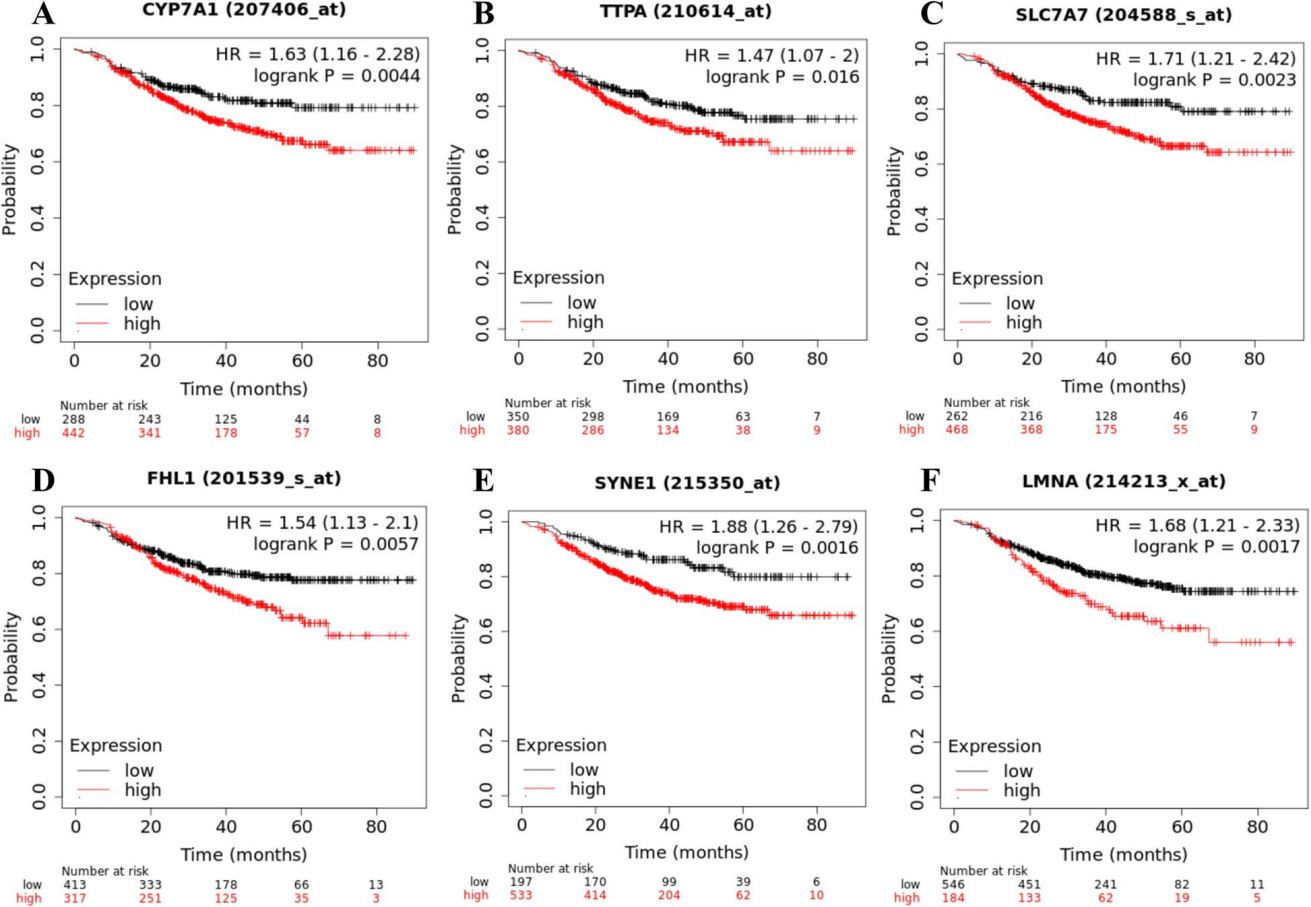
Genes related to elevated circulating LDL and relapse-free survival in the Kaplan Meier Plotter database Abbreviations: LDL, low-density lipoprotein

According to the GSEA database, a total of 31 genes (such as *CYP7A1*, *TTPA*, *SLC7A7*, *FHL1*, *SYNE1*, and *LMNA* etc.) related to the increase in LDL concentration in the circulating blood were identified. Analysis using the Kaplan–Meier Plotter database showed that the overexpression of these genes implied a worse RFS in breast cancer patients who received NAC (Fig. 9, also see supplementary Figure S4).

## Discussion

This is the first study to elucidate the influence of serum lipid fluctuations during NAC on the prognosis of patients with locally advanced breast cancer. Beyond that, this was the very first attempt to integrate the lipid metabolism index into Neo-Bioscore. The combination of local and systemic factors as well as tumor and host characteristics could stratify the prognosis of these patients more accurately.

This study first demonstrated that the relative increases in TC and LDL during the neoadjuvant period were independent prognostic risk factors for breast cancer patients, which indicated that changes in serum lipids, which were reportedly unfavorable to the cardiovascular system [23], also have a detrimental impact on survival outcomes in patients with breast cancer. However, the underlying mechanism is still open to investigation. This analysis of data from external database showed that several genes related to elevated circulating LDL were associated with prognosis. In addition, Nelson and colleagues reported that 27-hydroxycholesterol (27HC), the main metabolite of cholesterol, increased ER dependent tumor growth and enhanced liver X receptor (LXR) dependent metastasis in mouse models of breast cancer [24]. It was demonstrated subsequently that 27HC acted on immune myeloid cells at distal metastatic sites to create an immunosuppressive environment and helped to select cells that are resistant to ferroptosis, thus facilitating tumorigenicity and metastasis [25, 26]. These previous studies and the present external validation at least partially or indirectly supported the findings of this study from the front side. Conversely, the utilization of cholesterol-lowering drugs during endocrine therapy improved multiple survival outcomes, such as DFS, breast cancer free intervals, and distant recurrence free intervals, in patients with hormone receptor-positive breast cancer in the BIG1-98 trial [27], which partially supported the findings of this study from the reverse side.

The change in LDL level ranked first in terms of variable importance according to random forest algorithms. Several studies have shown that de novo lipogenesis increases the intracullular amounts of saturated and mono-unsaturated phospholipids intracellularly, thus resulting in a relative decrease in polyunsaturated acyl chains, which are susceptible to peroxidation, thereby protecting cells from cell death induced by reactive oxygen species (ROS)-related lipid peroxidation [28, 29]. Interestingly, LDL can also block these unfavorable polyunsaturated fatty acids in tumor cells [18], which may explain why increased LDL can act as a prognostic factor. Another promising prognosticator that is relevant to lipid metabolism is BMI, a variable that has been identified due to its high-ranking importance in the random survival forest. It is generally accepted that higher BMI is associated with the progression of multiple cancers [30, 31]. A retrospective meta-analysis revealed that increased BMI was linked to a greater risk of breast cancer, and the correlation was more intense in the Asia–Pacific population [6]. Moreover, several studies have demonstrated that breast cancer patients with overweight BMI turned out to have worse survival outcomes [32–35]. Compared with lipidomic analysis, BMI and serum LDL levels are readily available and highly feasible in clinical practice. The combination of the two parameters may provide a more comprehensive picture of lipid metabolism in vivo, which was successfully corroborated as a prognosticator for breast cancer patients undergoing NAC in this study.

Nowadays, metabolic reprogramming has been considered as one of the conspicuous hallmarks of cancer [12], especially regarding rewired lipid metabolism, which is the most prominent metabolic alteration in cancer [14]. Cancer cells are overdependent on fatty acids and cholesterol for rapid proliferation, division, invasion, metastasis, and membrane remodeling [13, 36, 37]. Moreover, lipid metabolism reprogramming also plays a crucial role in chemotherapy resistance and tumor immunology [17, 18]. Findings in this study illustrated better prognostic stratification of the updated prognostic model with the incorporation of the lipid metabolism index into Neo-Bioscore. After multidimensional assessment in both training and test sets, the updated model, which merged both intrinsic tumor characteristics and the host-specific lipid metabolism index, had superior predictive performance and clinical application. These data reinforced the prognostic significance of metabolic profiles in patients receiving NAC.

## Study strengths and limitations

The strength of this study lied in its originality and close relevance to clinical practice. Firstly, the updated model might serve as a conceptual paradigm to combine tumor characteristics and host metabolic profiles to assess the prognosis of breast cancer patients treated with NAC. Secondly, compared to other detection methods such as lipidomics, it is much easier and less costly to monitor serum lipids in the whole-course management of these patients.

This study had several limitations. Firstly, as a retrospective study based on prospective cohorts, the use of lipid-modifying medications during treatment is likely somewhat underestimated. Despite the fact that lipid levels at a certain time point may be affected by lipid-modifying drugs, this research explored the relationship between lipid fluctuations during the neoadjuvant period and prognosis to minimize the impact of specific individual differences on the results. Secondly, both the sample size and follow-up interval were not sufficient. However, this is an exploratory analysis for hypothesis generation based on a prospective cohort. A prospective study with a larger sample size and longer follow-up period is warranted for further verification.

## Conclusion

In conclusion, this study has not only identified a novel and reliable index of lipid metabolism as a prognostic indicator but also further optimized the prognostic stratification model by incorporating such an index for patients receiving NAC. The results of this study suggest that the medical staff should attach more importance to the management of serum lipids during neoadjuvant treatment. The findings of this study also lay a solid foundation for future in-depth explorations of lipid metabolism as an effective therapeutic target, thus providing new insights into the impact of host-specific factors on survival outcomes.

### Supplementary Information

Supplementary Material 1. 

Supplementary Material 2. 

Supplementary Material 3. 

Supplementary Material 4. 

Supplementary Material 5. 

## Data Availability

Data described in the manuscript will be made available upon reasonable request.

## Abbreviations

NAC: Neoadjuvant chemotherapy
DFS: Disease-free survival
RFS: Relapse-free survival
DRFS: Distance-recurrence-free survival
LRFS: Locoregional-recurrence-free survival
OS: Overall survival
TG: Triglyceride
TC: Total cholesterol
HDL: High-density lipoprotein
LDL: Low-density lipoprotein
NHDL: Non-high-density lipoprotein
BMI: Body mass index
pCR: Pathological complete response
TME: Tumor microenvironment
ROC: Receiver operating characteristic
AUC: Area under curve
DCA: Decision curve analysis
AIC: Akaike information criterion
IDI: Integrated Discrimination Improvement
ER: Estrogen receptor
HER2: Human epidermal growth factor receptor 2
