# Supplementary material for: Fluctuations in serum lipid levels during neoadjuvant treatment as novel predictive and prognostic biomarkers for locally advanced breast cancer: a retrospective analysis based on a prospective cohort
Source: Lipids Health Dis. 2024 Aug 22;23:261. doi: 10.1186/s12944-024-02140-x (PMC11340160; doi:10.1186/s12944-024-02140-x)
Supplement: Supplementary file 1 — Supplementary Material 1. [file 12944_2024_2140_MOESM1_ESM.docx]

**Supplementary Table S1. Distribution of BMI and lipid variations as categorical variables in training set and test set.**

| **Characteristic** | **Training set (N=120)** | **Test set (N=80)** | **P value** |
| --- | --- | --- | --- |
| **BMI ≤ 25.4kg/m^2^** | 97 (80.8%) | 65 (81.3%) | 0.941 |
| **BMI > 25.4kg/m^2^** | 23 (19.2%) | 15 (18.7%) |  |
| **TG change ≤ 1.51mmol/L** | 111 (92.5%) | 70 (87.5%) | 0.237 |
| **TG change > 1.51mmol/L** | 9 (7.5%) | 10 (12.5%) |  |
| **TC change ≤ -0.01mmol/L** | 72 (60.0%) | 47 (58.7%) | 0.860 |
| **TC change > -0.01mmol/L** | 48 (40.0%) | 33 (41.3%) |  |
| **HDL change ≤ -0.51mmol/L** | 83 (69.2%) | 56 (70.0%) | 0.900 |
| **HDL change > -0.51mmol/L** | 37 (30.8%) | 24 (30.0%) |  |
| **LDL change ≤ 0.54mmol/L** | 99 (82.5%) | 69 (86.3%) | 0.479 |
| **LDL change > 0.54mmol/L** | 21 (17.5%) | 11 (13.7%) |  |

Abbreviations: BMI, body mass index; TG, triglyceride; TC, total cholesterol; HDL, high-density lipoprotein; LDL, low-density lipoprotein; NHDL, non-high-density lipoprotein.

**Supplementary Table S2. Number of events for different outcomes in training set and test set.**

| **Outcome** | **Training set (N=120)** | **Test set (N=80)** |
| --- | --- | --- |
| **pCR (N)** | 28 | 18 |
| **DFS events (N)** | 14 | 10 |
| **RFS events (N)** | 12 | 8 |
| **DRFS events (N)** | 12 | 8 |
| **LRFS events (N)** | 8 | 4 |
| **OS events (N)** | 7 | 4 |

Abbreviations: pCR, pathological complete response; DFS, disease-free survival; RFS, relapse-free survival; DRFS, distance-recurrence-free survival; LRFS, local-recurrence-free survival; OS, overall survival.

**Supplementary Table S3. The univariate and multivariate analysis of pre-NAC TC in estimating pCR.**

| **Characteristic** | **Total (N)** | **Univariate analysis** | | **Multivariate analysis** | |
| --- | --- | --- | --- | --- | --- |
|  |  | OR (95%CI) | P value | OR (95%CI) | P value |
| **lower pre-NAC TC** | 98 | Reference |  | Reference |  |
| **higher pre-NAC TC** | 22 | 2.879 (1.073 - 7.720) | 0.036 | 3.033 (1.031 - 8.923) | 0.044 |
| **hormone receptor-negative** | 44 | Reference |  | Reference |  |
| **hormone receptor-positive** | 76 | 0.328 (0.137 - 0.783) | 0.012 | 0.380 (0.148 - 0.977) | 0.045 |
| **HER2-negative** | 79 | Reference |  | Reference |  |
| **HER2-positive** | 41 | 1.629 (0.684 - 3.882) | 0.270 | 1.800 (0.695 - 4.662) | 0.226 |
| **clinical stage II** | 32 | Reference |  | Reference |  |
| **clinical stage III** | 88 | 0.704 (0.280 - 1.771) | 0.455 | 0.572 (0.204 – 1.607) | 0.289 |
| **Ki-67 ≤ 30%** | 52 | Reference |  | Reference |  |
| **Ki-67 > 30%** | 68 | 4.804 (1.681 - 13.727) | 0.003 | 4.236 (1.407 - 12.754) | 0.010 |

Abbreviations: NAC, neoadjuvant chemotherapy; pCR, pathological complete response; TC, total cholesterol; HER2, human epidermal growth factor receptor 2; OR, odds ratio; CI, confidence interval.

**Supplementary Table S4. The univariate and multivariate analysis of TC fluctuation during NAC in estimating pCR.**

| **Characteristic** | **Total (N)** | **Univariate analysis** | | **Multivariate analysis** | |
| --- | --- | --- | --- | --- | --- |
|  |  | OR (95%CI) | P value | OR (95%CI) | P value |
| **TC relative decrease** | 72 | Reference |  | Reference |  |
| **TC relative increase** | 48 | 0.248 (0.087 - 0.708) | 0.009 | 0.300 (0.098 - 0.916) | 0.035 |
| **hormone receptor-negative** | 44 | Reference |  | Reference |  |
| **hormone receptor-positive** | 76 | 0.328 (0.137 - 0.783) | 0.012 | 0.431 (0.168 - 1.105) | 0.080 |
| **HER2-negative** | 79 | Reference |  | Reference |  |
| **HER2-positive** | 41 | 1.629 (0.684 - 3.882) | 0.270 | 1.475 (0.565 – 3.847) | 0.427 |
| **clinical stage II** | 32 | Reference |  | Reference |  |
| **clinical stage III** | 88 | 0.704 (0.280 - 1.771) | 0.455 | 0.558 (0.200 – 1.557) | 0.266 |
| **Ki-67 ≤ 30%** | 52 | Reference |  | Reference |  |
| **Ki-67 > 30%** | 68 | 4.804 (1.681 - 13.727) | 0.003 | 4.153 (1.374 - 12.550) | 0.012 |

Abbreviations: NAC, neoadjuvant chemotherapy; pCR, pathological complete response; TC, total cholesterol; HER2, human epidermal growth factor receptor 2; OR, odds ratio; CI, confidence interval.

**Supplementary Figure S1. Relationship of baseline (pre-NAC) TG, TC, HDL and LDL with DFS (A-D) and RFS (E-H).**

**
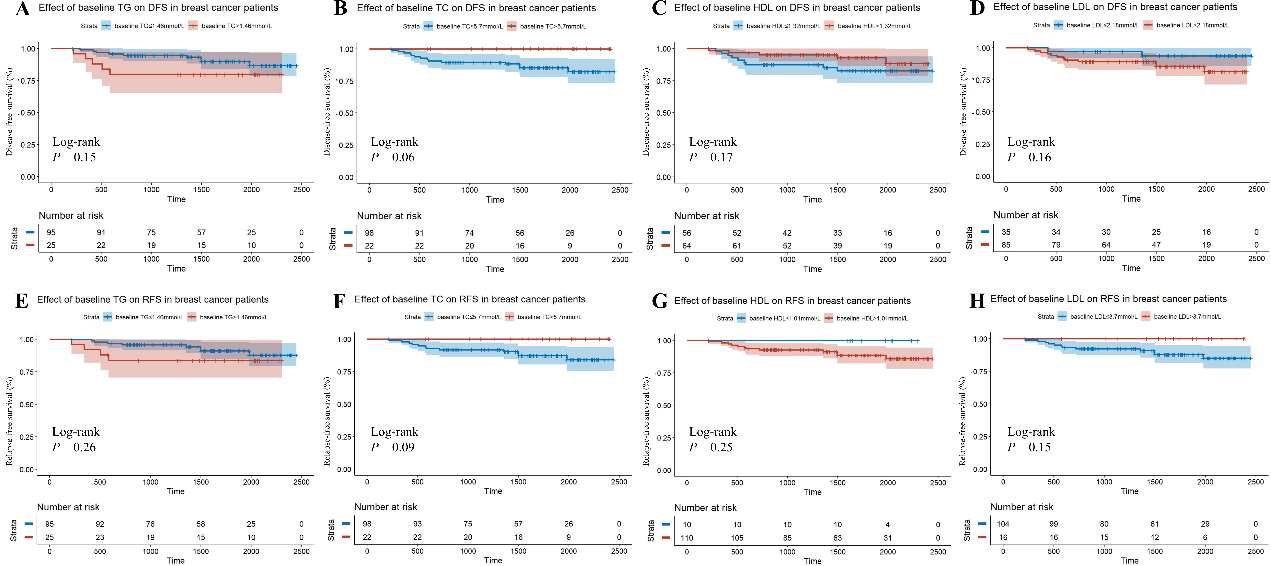
**

Abbreviations: DFS, disease-free survival; RFS, relapse-free survival; TG, triglyceride; TC, total cholesterol; HDL, high-density lipoprotein; LDL, low-density lipoprotein.

**Supplementary Figure S2. Relationship of preoperative (post-NAC) TG, TC, HDL and LDL with DFS (A-D) and RFS (E-H).**

**
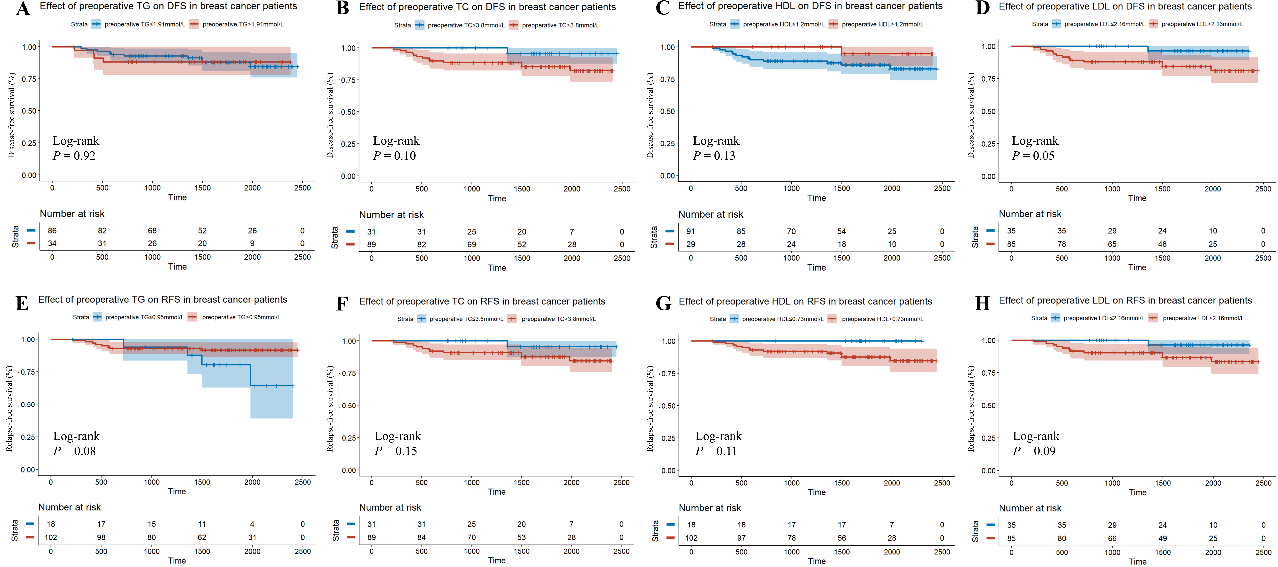
**

Abbreviations: DFS, disease-free survival; RFS, relapse-free survival; TG, triglyceride; TC, total cholesterol; HDL, high-density lipoprotein; LDL, low-density lipoprotein.

**Supplementary Figure S3. Relationship of TG, TC and HDL fluctuations during NAC with DFS (A-C) and RFS (D-F).**

**
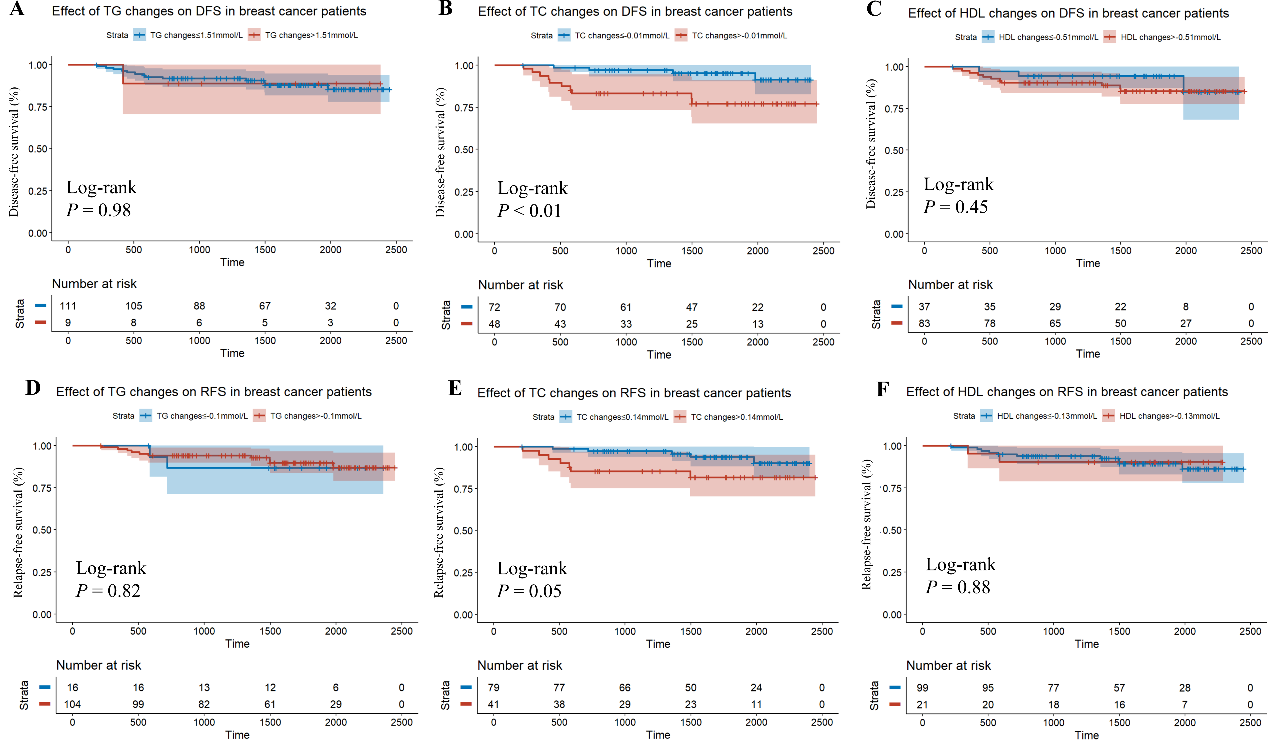
**

Abbreviations: DFS, disease-free survival; RFS, relapse-free survival; TG, triglyceride; TC, total cholesterol; HDL, high-density lipoprotein; NAC, neoadjuvant chemotherapy.

**Supplementary Figure S4. Other genes related to elevated circulating LDL and RFS in the Kaplan-Meier Plotter database.**

**
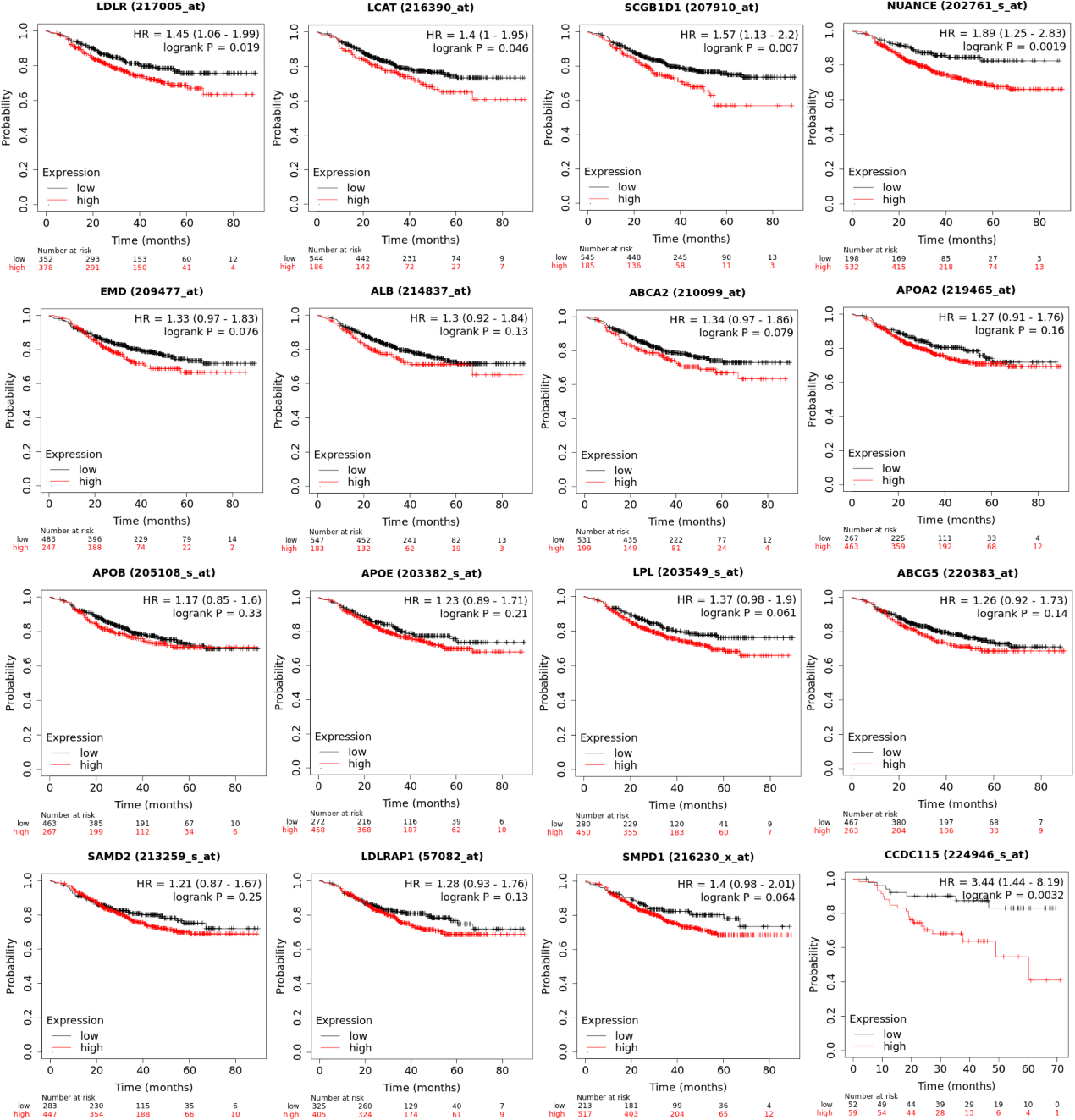
**

Abbreviations: LDL, low-density lipoprotein.
