# Supplementary figures and images for: Fluctuations in serum lipid levels during neoadjuvant treatment as novel predictive and prognostic biomarkers for locally advanced breast cancer: a retrospective analysis based on a prospective cohort
Source: Lipids Health Dis. 2024 Aug 22;23:261. doi: 10.1186/s12944-024-02140-x (PMC11340160; doi:10.1186/s12944-024-02140-x)

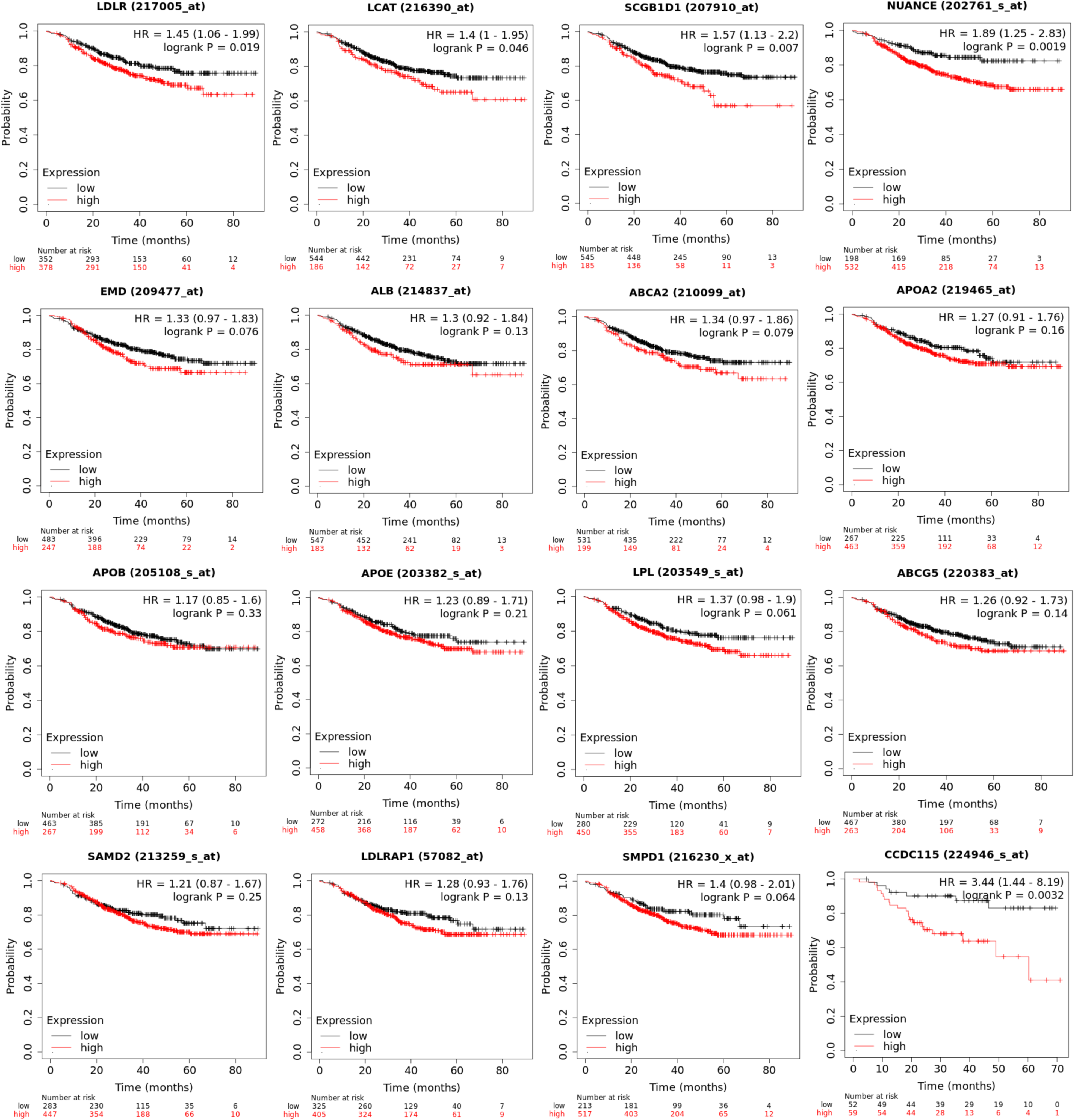

Supplement: Supplementary file 5 — Supplementary Material 5. [file 12944_2024_2140_MOESM5_ESM.tif]
